# Beyond speech: Exploring diversity in the human voice

**DOI:** 10.1016/j.isci.2023.108204

**Published:** 2023-10-14

**Authors:** Andrey Anikin, Valentina Canessa-Pollard, Katarzyna Pisanski, Mathilde Massenet, David Reby

**Affiliations:** 1 Division of Cognitive Science, Lund University, Lund, Sweden; 2 ENES Bioacoustics Research Lab, CRNL, University of Saint-Etienne, CNRS, Inserm, 23 rue Michelon, 42023 Saint-Etienne, France; 3 Psychology, Institute of Psychology, Business and Human Sciences, University of Chichester, Chichester, West Sussex PO19 6PE, UK; 4 CNRS French National Centre for Scientific Research, DDL Dynamics of Language Lab, University of Lyon 2, 69007 Lyon, France; 5 Institute of Psychology, University of Wrocław, Dawida 1, 50-527 Wrocław, Poland

**Keywords:** Natural sciences, Biological sciences, Evolutionary biology

## Abstract

Humans have evolved voluntary control over vocal production for speaking and singing, while preserving the phylogenetically older system of spontaneous nonverbal vocalizations such as laughs and screams. To test for systematic acoustic differences between these vocal domains, we analyzed a broad, cross-cultural corpus representing over 2 h of speech, singing, and nonverbal vocalizations. We show that, while speech is relatively low-pitched and tonal with mostly regular phonation, singing and especially nonverbal vocalizations vary enormously in pitch and often display harsh-sounding, irregular phonation owing to nonlinear phenomena. The evolution of complex supralaryngeal articulatory spectro-temporal modulation has been critical for speech, yet has not significantly constrained laryngeal source modulation. In contrast, articulation is very limited in nonverbal vocalizations, which predominantly contain minimally articulated open vowels and rapid temporal modulation in the roughness range. We infer that vocal source modulation works best for conveying affect, while vocal filter modulation mainly facilitates semantic communication.

## Highlights

• Speech, singing, and nonverbal vocalizations are three distinct vocal domains
• Voice pitch is low and stable in speech compared to nonverbal vocalizations
• Nonverbal vocalizations are poorly articulated and mostly contain a-like vowels
• Source modulation is critical for conveying affect, filter modulation for semantics

## Graphical abstract

**Figure:**
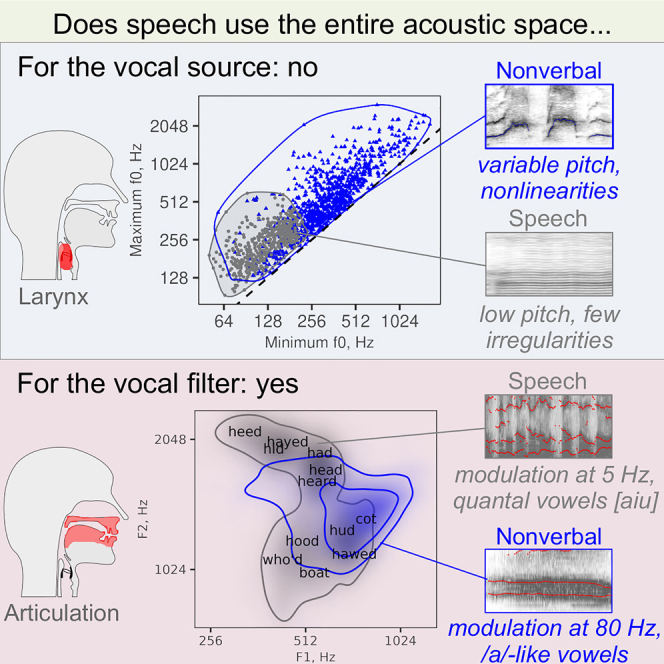

## Introduction

An alien “armchair anthropologist” studying human vocal communication from the comfort of a space station might do a quick scan for scientific papers, leaf through a textbook or two on phonetics, and conclude that all that humans do with their voices is speak. Indeed, spoken language has been studied so extensively for a good reason: it is a uniquely human ability of crucial social importance.^1^ Yet, a day or two of fieldwork in any human society would reveal an unsuspected profusion of vocal acrobatics that sound nothing like speech: squeals of delight and groans of agony,^2^ beatboxing and throat singing,^3^ whistled languages^4^ and competitions for the best seagull imitation (http://www.gullscreeching.eu/). Speech, singing, and emotional nonverbal vocalizations (e.g., laughs and screams) may represent three vocal domains: the “vocal instrument” is the same, but the functions and “design requirements” shaping the form of these vocal behaviors may differ. However, we still do not know the extent to which these acoustic domains overlap: do they occupy different subregions of the acoustic space available to us, and if so, what makes each subregion special?

To answer this question, we collected an international corpus of neutral speech from 23 languages (n = 416, 42 min of audio, 23 languages), non-neutral speech (n = 200, 9 min, including emotional speech, cross-gender imitation, public oratory, and pet-directed speech), singing (n = 128, 30 min, including chants, classical singing, folk, lullabies, pop, rock, and unconventional techniques such as Tuvan and Tibetan throat singing), and nonverbal vocalizations (n = 969, 29 min, including laughs, cries, screams, imitated animal calls, etc.; see STAR Methods and Table 1 for more details). Together, these cover the vast variety of voiced sounds produced by adult humans in various cultures, including non-Western ones. We did not consider infant cries or non-phonated sounds that do not involve the vocal folds (e.g., whistles). All 1,745 audio recordings were analyzed acoustically with R package *soundgen*.^5^

**Table 1:** Analyzed audio recordings

Category	Subcategory	N stimuli (male/female)	Duration voiced, min
Neutral speech	Neutral speech	416 (217/199)	42.1
Non-neutral speech	Emotional speech	66 (35/31)	2
	Gender imitation	96 (45/51)	2.2
	Oratory	13 (6/7)	4.1
	Pet-directed speech	25 (13/12)	0.4
	SUBTOTAL	200 (99/101)	8.7
Singing	Chants	34 (17/17)	8.7
	Classical	18 (7/11)	3.7
	Folk	19 (11/8)	4.5
	Lullabies	12 (9/3)	2.9
	Pop	31 (20/11)	7.4
	Rock	26 (11/15)	6.5
	Unconventional singing	20 (6/14)	4.6
	SUBTOTAL	160 (81/79)	38.4
Nonverbal	Animal imitation	60 (32/28)	6.7
	Cry	45 (22/23)	1.9
	Grunt	45 (22/23)	0.4
	Laugh	275 (150/125)	6.2
	Moan	118 (58/60)	3.3
	Roar	187 (55/132)	4.2
	Scream	214 (158/56)	5.7
	Sigh	25 (10/15)	0.2
	SUBTOTAL	969 (507/462)	28.5
	TOTAL	1745 (904/841)	117.8

Our analyses follow the tenets of the acoustic source-filter theory,^6^^,^^7^ which helps to understand the acoustic variability of vocalizations in terms of their mechanisms of production. *Source* stands for the source of acoustic excitation, here restricted to vocal fold vibration and hereafter termed phonation. When the vocal folds vibrate in a periodic way, the spectrum of the source signal is characterized by a fundamental frequency (*f*_o_) and its harmonics, which are integer multiples of the fundamental frequency. The *f*_o_ corresponds to the number of vibrations of the vocal folds per second and is responsible for the perceived *pitch* of the signal. This sound produced by the vocal folds (phonated source) then passes through the supralaryngeal vocal tract (oral and/or nasal cavities), which together act as an acoustic *filter*. Some frequency bands are preferentially absorbed by this filter, whereas others pass with little attenuation, creating spectral peaks and valleys. Prominent spectral peaks that correspond to vocal tract resonances are known in phonetics as *formants* and affect the timbre of the radiated voice, particularly with regard to the perceived vowel quality.

Crucially, in our acoustic analyses *f*_o_ contours were extracted manually, ensuring accurate measurements not only of central tendency, but also of *f*_o_ range and slope. All deviations from regular phonation (nonlinear phenomena), which typically give vocal signals a perceptually rough or harsh acoustic quality, were annotated manually. Nonlinear phenomena included sudden frequency jumps, period doubling or tripling (subharmonics, as in throat singing), amplitude modulation by supralaryngeal structures creating vocal roughness (as in Louis Armstrong’s voice), deterministic chaos (irregular vibration of vocal folds, as in very rough roars), and vocal fry (a low-pitched, quiet, creaky voice with irregular glottal cycles). We also measured formant frequencies in vowel-like regions in nonverbal vocalizations (see STAR Methods) to compare their distributions to the well-understood formant modulations in the actual vowels found in speech.^6^^,^^7^ Finally, all sections of all recordings were used to generate and compare spectro-temporal modulation spectra.

On the basis of these analyses, we aimed at characterizing the acoustic diversity of nonverbal vocalizations, singing, and speech by contrasting several acoustic dimensions: source modulation (the range and variability of *f*_o_, occurrence of nonlinear phenomena), vowel quality defined by the first two formant frequencies (*F1/F2*), and joint spectro-temporal modulation. Based on the principle of form-function correspondence in acoustic communication,^8^^,^^9^ we expected that speech would be characterized by a relatively low and stable source (less variable intonation and limited nonlinear phenomena) and a more varied filter (a wider *F1/F2* space) than nonverbal vocalizations, with non-neutral speech and singing occupying intermediate positions.

## Results

### Speech has low pitch and few nonlinear phenomena

**Figure 1:**
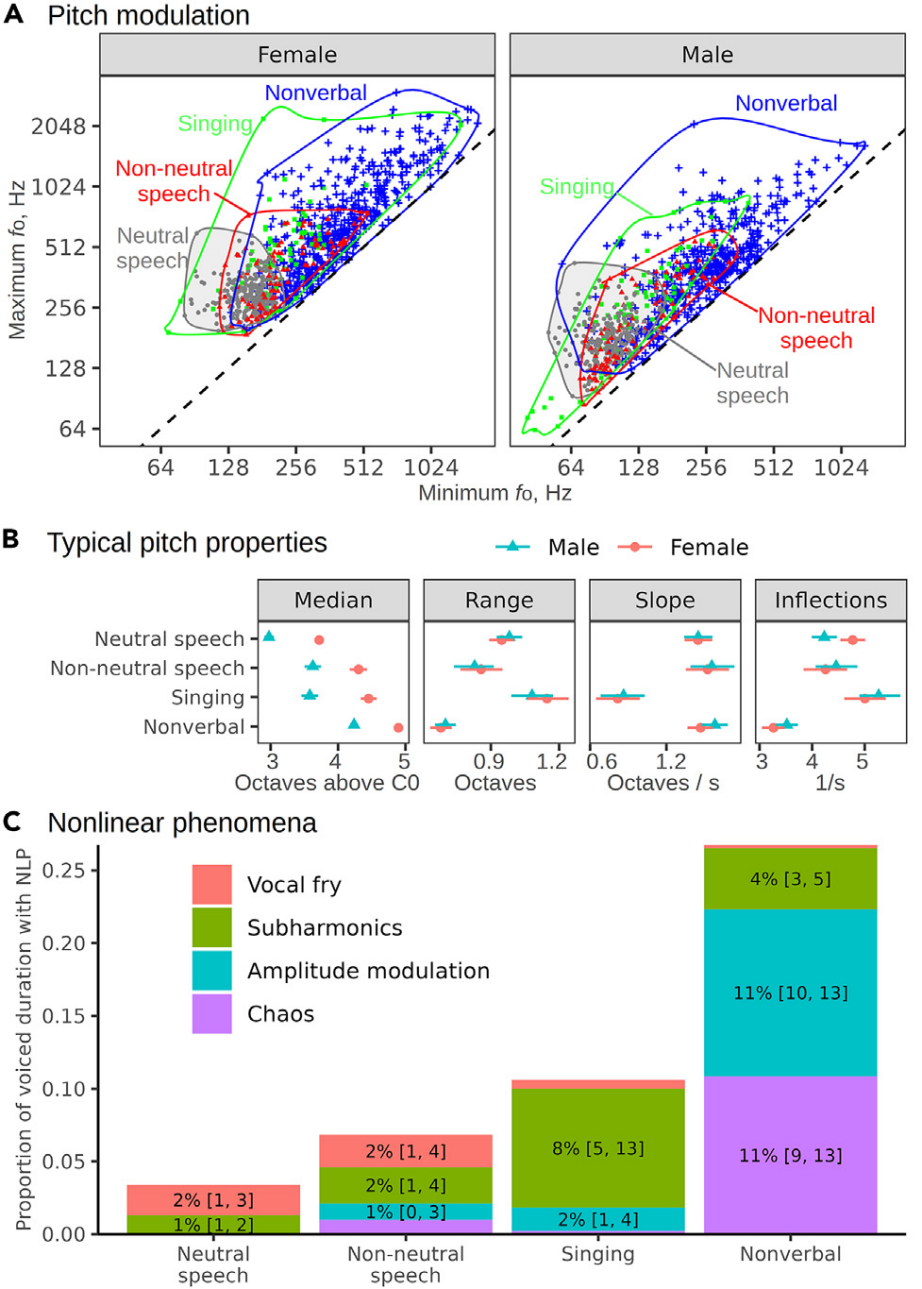
Vocal source properties in speech, singing, and nonverbal vocalizations (A) Neutral speech occupies a small subregion of anatomically possible pitch modulation, shown here as scatterplots of minimum by maximum *f*_o_ values per recording, separately for male and female speakers. Contours enclose the entire observed range within each category and sex. (B) Typical values of voice pitch descriptives vary among speech, singing and nonverbal vocalizations: fitted values from mixed models (medians of posterior distribution and 95% CI). Median = median *f*_o_ in octaves above C0 (16 Hz); range = *f*_o_ range, octaves; slope = mean absolute slope of *f*_o_, octaves/s; inflections = number of *f*_o_ inflections per second. (C) Typical proportions of voiced frames affected by various nonlinear phenomena are nearly ten times higher in nonverbal vocalizations compared to neutral speech (medians of posterior distributions and 95% CI shown for the most common types).

To begin with the vocal source, our results show that speech occupies a small fraction of the available *f*_o_ space, stopping about two octaves below the upper limit reached in singing and nonverbal vocalizations (Figure 1A). To obtain a more nuanced view, we modeled typical values of different pitch descriptives in different categories with Bayesian mixed models (Figure 1B). Neutral speech had the lowest median *f*_o_ (men 129 Hz, 95% CI [123, 135], women 215 Hz [205, 226]), followed by non-neutral speech such as public oratory (men 202 Hz [185, 219], women 323 Hz [295, 352]). Both singing and nonverbal vocalizations displayed a lot of pitch variability: median *f*_o_ per recording reached up to 1 kHz in men and 2 kHz in women, with typical values about an octave above the speech range (Figure 1B). Of course, some extreme values may come from vocally exceptional individuals, such as famous singers, but even ordinary speakers in the *nonverbal* category routinely screamed 1–2 octaves above the normal speech register within each sex. The typical amount of *f*_o_ variability per recording was more comparable, close to one octave in both neutral and non-neutral speech and singing and dropping to about 0.7 octaves in the shorter nonverbal vocalizations (Figure 1B). Singing also had the smallest average absolute *f*_o_ slope (men 0.9 [0.7, 1.0] oct/s, women 0.8 [0.7, 1.0] oct/s) and the greatest number of *f*_o_ inflections per second (men 5.3 [4.9, 5.7], women 5.0 [4.6, 5.4]), consistent with singing long and steady notes with a shallow, but rapid vibrato.

Overall, speech was fairly distinct in terms of its melodic contours. A Random Forest classifier using speaker sex and just four pitch descriptives (*f*_o_ median, range, slope, and inflections per second) had an out-of-sample accuracy of 71% when classifying neutral speech, non-neutral speech, singing, and nonverbal vocalizations (chance level = 1/4 = 25%). This rose to 84% if non-neutral and neutral speech were treated as a single speech category (chance level = 33%). Speech also lacked the perceptual harshness of other vocal sounds. Nonlinear vocal phenomena were nearly absent in speech, apart from occasional subharmonics (1.3% [0.8, 2.1] of voiced frames in neutral speech and 4.2% [3.3, 5.2] in non-neutral speech) and vocal fry (2.1% [1.4, 3.0] in neutral speech). In contrast, nonverbal vocalizations often contained high proportions of these harsh-sounding nonlinear phenomena, especially amplitude modulation (11.5% [9.9, 13.2] of voiced frames) and deterministic chaos (10.9% [9.3, 12.6] of voiced frames), both of which were essentially absent from neutral speech and found in only 1% of voiced frames in non-neutral—usually angry—speech (Figure 1C). Sudden frequency jumps were also much more common in nonverbal vocalizations (3.0 jumps per minute, 95% CI [2.2, 4.0]) than in speech (0.18 [0.09, 0.35]). Pitch jumps were also nearly absent in singing (0.06 [0.01, 0.21]), where they are known as voice cracks and carefully avoided in most musical genres. Thus, singing and especially nonverbal vocalizations display far less regular phonation compared to speech.

### Formant patterns vary more in speech than in nonverbal vocalizations

**Figure 2:**
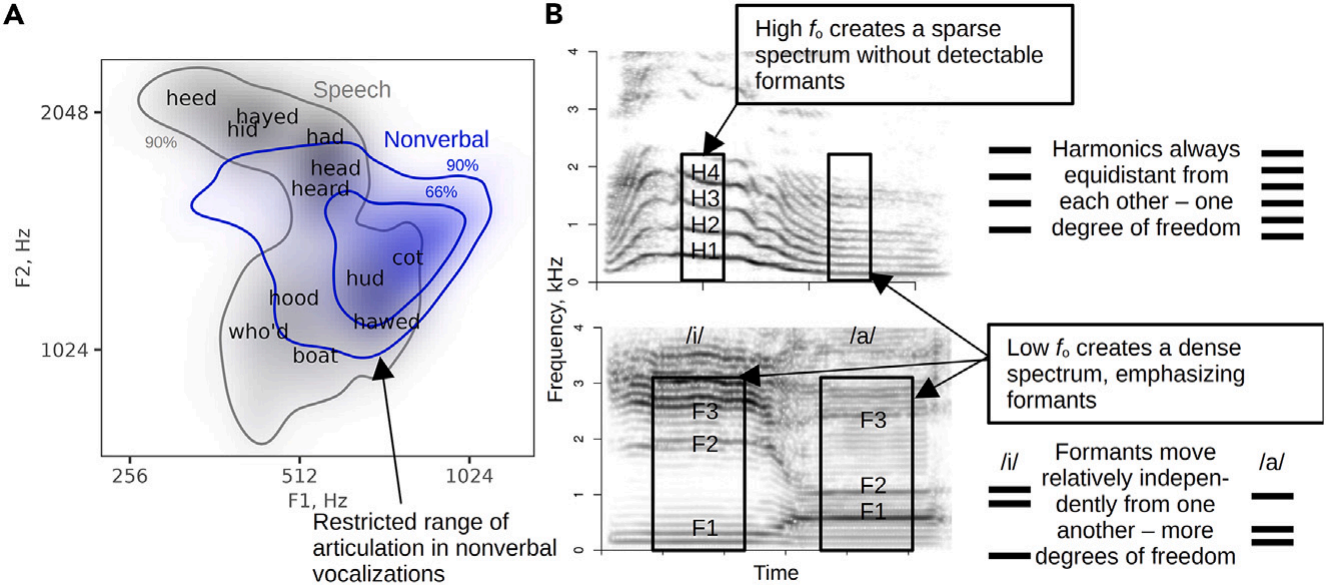
Filter properties in speech and nonverbal vocalizations (A) Nonverbal vocalizations mostly contain open vowels, especially [a]. Color gradients show distribution densities for vowels in speech (gray, data taken from Hillenbrand’s corpus^10^) and in nonverbal vocalizations (blue). The text labels correspond to vowel centroids, while contour lines show the areas containing different proportions of observations. Formants F1 and F2 are normalized to an apparent vocal tract length of 17 cm to make the formant space sex- and speaker-size-invariant. (B) Formants can be thought of as bar codes capable of encoding more information than does voice pitch. Spectrograms of a nonverbal vocalization (above) and two vowels by male speakers produced with Gaussian windows of 40 ms and 25 ms, respectively. Harmonics of *f*_o_ are redundant in the sense that a single number (*f*_o_) encodes the location of all spectral peaks (note the parallel harmonic tracks in the first vocalization). In contrast, formant frequencies can encode more information than *f*_o_ does because they can vary relatively independently (note the non-parallel formant tracks in the vowels), and such variation is meaningful.

Moving on from the source to the filter, we manually measured the frequencies of the first four formants in vowel-like regions of 625 nonverbal vocalizations. The remaining 284 vocalizations were either too high-pitched for formant analysis (see STAR Methods) or did not contain sufficiently long and stable vowel-like regions, leading to the exclusion of two-thirds of screams and about 10–25% of other types of nonverbal vocalizations from the formant analysis. The measured frequencies of formants F1 and F2 were normalized to take into account vocal tract length (see STAR Methods) and then compared to similarly normalized F1-F2 in a large published corpus of American vowels.^10^ Our results show that vowels in nonverbal vocalizations closely clustered around the schwa and a-like vowels (Figure 2A). The other two quantal vowels, [i] and [u], were very rare in nonverbal vocalizations. In contrast, and in line with an earlier observation that F1 can be very high in laughs,^11^ we found many open vowels with an extremely high F1, suggesting that the mouth was opened very wide during their production,^7^^,^^12^ presumably to be as loud as possible.^13^ A similar phenomenon is reported in soprano singers, who intentionally adjust their mouth openings to lock F1 to *f*_o_ or to one of its harmonics.^12^ This is also sometimes observed in non-human animals such as singing gibbons.^14^ Apart from that, we found that speakers articulated very little when producing nonverbal vocalizations, or at least formant configurations in vowel-like regions showed limited deviation from the schwa or a-like vowels, suggesting a physically relaxed vocal tract with little or no attempt to articulate. In contrast, the vowel space of speech covered the entire region shown in Figure 2A (with more granular divisions in languages like German or Swedish), even without taking into account formant transitions in diphthongs and glides like [j] and hyper-articulated speech, which would push the boundaries of the formant space even further.^15^

### Differences between vocal domains are captured by the modulation spectrum

**Figure 3:**
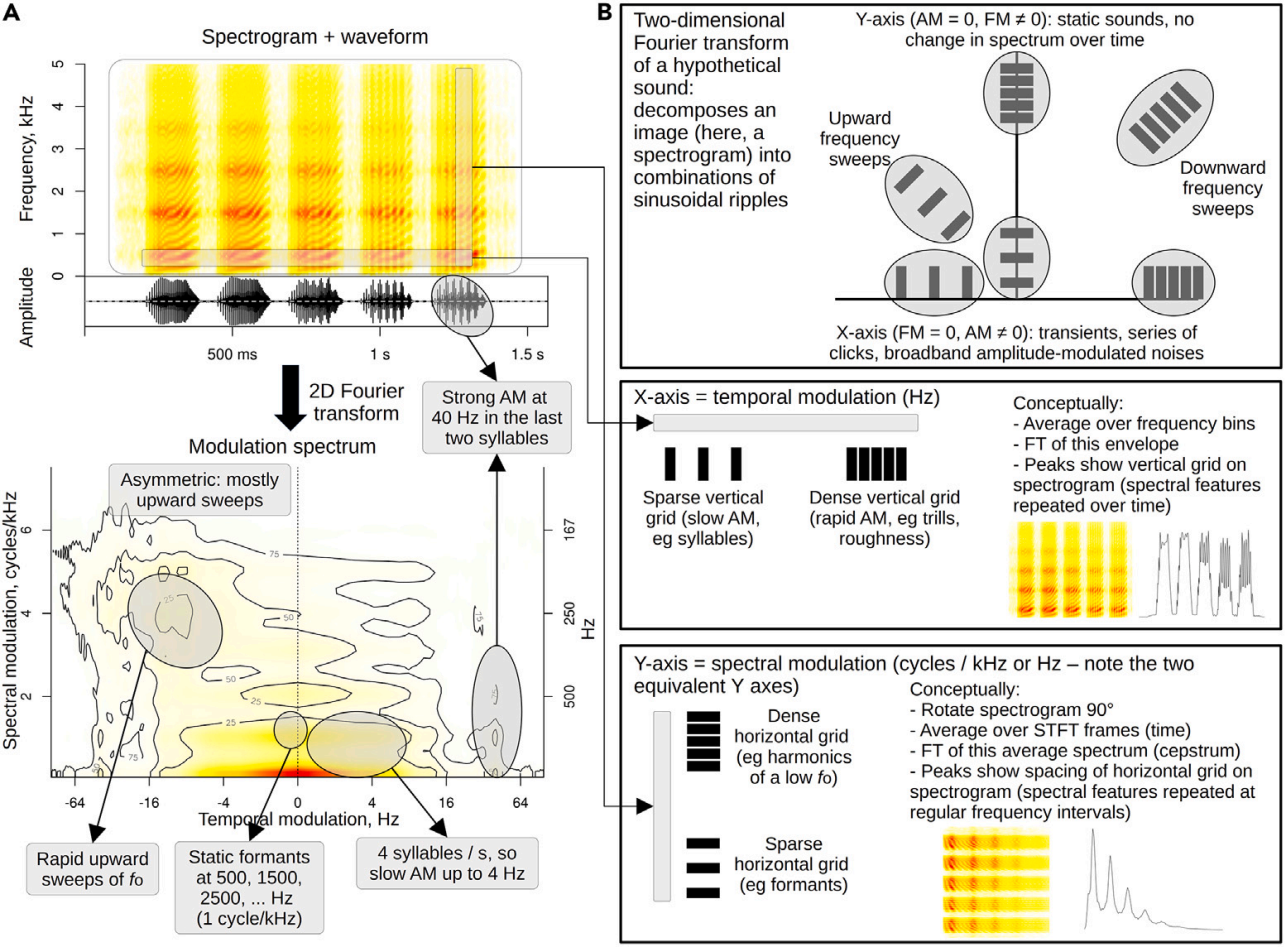
The spectro-temporal modulation spectrum (A) A synthetic laugh-like sound created with *soundgen*,^5^ which has upward *f*o contours (100–300 Hz) in every syllable, static equidistant formants (500 Hz, 1500 Hz, …), and amplitude modulation at 40 Hz in the last few syllables. This same sound is shown as a spectrogram with its corresponding waveform below, and then as a modulation spectrum created with *soundgen::modulationSpectrum* using a window length of 15 ms and a step of 5 ms. (B) A conceptual illustration of the nature of a modulation spectrum. Treating the spectrogram as an image, the modulation spectrum represents it as a combination of horizontal, vertical, and slanting ripples or grids with different spacings, which acoustically correspond to regularly repeated spectro-temporal patterns. AM = amplitude modulation, FM = frequency modulation, FT = Fourier transform, STFT = short-time Fourier transform.

A third way to compare speech with other vocal behaviors is to consider the joint spectro-temporal modulation spectrum.^16^^,^^17^ As explained in the STAR Methods and shown schematically in Figure 3, this technique decomposes a conventional spectrogram into its modulation components along the time and frequency dimensions, capturing variability in both the source and the filter.^16^^,^^17^

**Figure 4:**
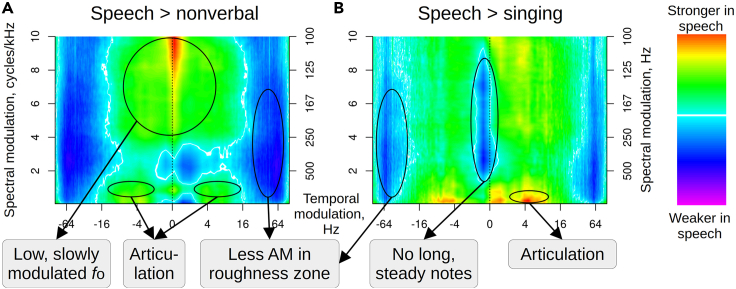
Differences between spectro-temporal modulation spectra of speech and nonverbal vocalizations Log-ratios of normalized modulation spectra averaged within each category (see STAR Methods). For instance, speech has pronounced articulation-related amplitude modulation under 20 Hz compared to nonverbal vocalizations (yellow and red), while nonverbal vocalizations have strong modulation in the roughness zone above 50 Hz (blue).

Comparing the average modulation spectrum of all speech recordings with that of all nonverbal vocalizations (Figure 4A), we show that speech has more low-frequency (100–200 Hz) spectral components modulated at a relatively slow rate of 0–16 Hz, reflecting the low *f*_o_ and relatively stable voice pitch in speech. Speech also has more temporal modulation at about 3–8 Hz with a peak at 5 Hz, which corresponds to syllable articulation.^18^^,^^19^^,^^20^ On the contrary, nonverbal vocalizations display more spectral modulation between 200 and 800 Hz, caused by *f*_o_ being much higher in nonverbal vocalizations vs. speech, as well as rapid temporal modulation peaking at about 65 Hz. This fast amplitude modulation is responsible for the harsh or rough perceptual character of many nonverbal vocalizations. It has previously been shown to extend well up to 200 Hz in particularly rough screams,^21^ but the peak between 50 and 70 Hz observed here falls right in the middle of the perceptual roughness zone.^22^ Slower temporal modulation is present in some nonverbal vocalizations, notably laughs with their syllable-like rhythm at about 5 Hz,^23^ but most nonverbal vocalizations appear to lack a well-defined temporal structure. The modulation spectrum of singing shows an abundance of steady tones with weaker slow temporal modulation and more roughness compared to speech, which is consistent with the recent results of Albouy et al.^24^ (Figure 4B). In sum, spectro-temporal modulation spectra reveal systematic differences between the three vocal domains, including both the vocal source (i.e., high *f*_o_ and vocal roughness in nonverbal vocalizations, steady notes in singing) and the filter (more articulation in speech than in singing and especially in nonverbal vocalizations).

## Discussion

Our systematic and large-scale acoustic comparison confirmed what is intuitively familiar: speech, singing, and nonverbal vocalizations are three very different ways of using the human voice. Obviously, there are intermediate forms: recitativo and rap singing, conventionalized speech-like interjections like *Ouch* and *Huh?*,^25^^,^^26^ and indeed emotional speech itself, which borrows prosodic markers from our nonverbal repertoire.^1^ Overall, however, these three vocal domains are distinct acoustically. While the systematic differences between voice modulation in speech and singing are increasingly recognized,^20^^,^^24^ here we point out that nonverbal vocalizations represent a third distinct category, which is particularly interesting because it offers a window to our evolutionary past.^27^ Comparing these vocal domains shows how our voices have adapted to the task of efficiently encoding complex semantic information: supralaryngeal articulation has exploded with the advent of speech, while the full potential of vocal source modulation is only revealed in nonverbal communication and vocal arts.

We confirm that the phonated vocal source in speech is confined to a narrow region of relatively low-pitched acoustic excitation with few nonlinear phenomena compared to the full physiological vocal range. Speech intonation is by no means flat: as a crucial component of speech prosody, *f*_o_ variation conveys both linguistic (e.g., syllable stress and lexical tones) and para-linguistic (e.g., irony and emotion) information.^28^ Furthermore, some variability in *f*_o_ can be very useful for making the formants easier to detect, particularly in relatively high-pitched female voices.^29^ Still, the range of *f*_o_ variation in ordinary speech is limited compared to what we find in both nonverbal vocalizations and singing (Figure 1). For instance, *f*_o_ sweeps in lexical tones span about an octave^30^^,^^31^—just enough to make the tones easily distinguishable without wasting energy or jeopardizing vocal health. According to our results, non-neutral speech and especially singing do venture beyond this low-pitched tonal region, but the full potential of the vocal source is only explored in nonverbal vocalizations, which include both a very broad pitch range and a rich variety of nonlinear phenomena. One reason for limited phonated source variability in speech is that the reliability of communication is weighed against its metabolic cost, according to Zipf’s principle of least effort.^32^ Loud, rough, high-pitched vocalizations, such as screams or aggressive roars, require high lung pressure and extreme tension in the vocal folds, which is metabolically costly and carries high risk of vocal injury.^7^ While their high salience^21^^,^^33^ and potential for honest signaling of affective states^34^^,^^35^^,^^36^ are advantageous in emotionally charged contexts, extreme source modulation would be excessively costly and inefficient for semantic communication. In addition, a low *f*_o_ is optimal for speech because it creates a spectrally dense sound source, which emphasizes the formants.^8^ The high notes reached in screams and soprano singing are artistically and emotionally expressive, but the spectrum is too sparse to allow vocal tract resonance frequencies to be resolvable, making speech at such frequencies nearly unintelligible.

As for nonlinear phenomena, it has been suggested that the loss of vocal membranes and air sacks in the course of human evolution was necessary to provide a stable vocal source for speech.^8^ However, our results highlight the fact that the ability to produce irregular, strongly modulated phonation is not lost in modern humans, but perhaps only brought under better control as nonlinearities remain plentiful in nonverbal vocalizations. Thus, our data suggest that the source stability discussed by Nishimura et al.^8^ is not a characteristic of the human voice in general, but rather a speech-specific feature. This indicates that humans may have retained, or even improved upon, the ability to produce a very wide range of highly diverse and modulated, phonated sounds, including modulation of voice pitch and nonlinear phenomena, which we utilize for a range of vocal behaviors. Importantly, also, the fact that humans do not exploit this vast acoustic space for speech, even though we easily could, lends support to the prediction that excessive source variability is either inefficient for encoding semantic information or even detrimental to formant and thus speech perception, a prediction that remains to be tested.

In contrast to the limited range of vocal source modulation, articulation is absolutely central to speech. In acoustic terms, this means that speech has very pronounced spectro-temporal modulation associated with syllable patterning and formant transitions. In contrast, articulation in nonverbal vocalizations is relatively limited, apart from F1 occasionally reaching very high frequencies, presumably as a side effect of vocalizing loudly with a wide-open mouth.^37^ Why does speech rely so strongly on articulation? We suggest that quasi-independent modulation of individual formants offers more degrees of freedom compared to modulating harmonic spacing (Figure 2B), greatly enhancing the information-carrying potential of the speech signal. Compared to music, speech may be more resistant to spectral than to temporal degradation,^38^ but other reports suggest that intelligibility is preserved when either only temporal or only spectral modulations are removed.^17^ The notion that a communication channel that is greatly impoverished compared to speech can still support rich and speech-like communication is corroborated by the existence of drummed and whistled languages, which rely almost exclusively on rhythm^39^ and a single spectral component like F2,^4^ respectively. The speech channel is designed to be redundant, and different elements contribute to encoding semantic information: static formant patterns in vowels, formant transitions in diphthongs and consonants, transients like tongue clicks, and the overall speech rhythm. All of these features depend on rapid and precise cortical control of supralaryngeal articulators—the lips, tongue, lower jaw, and velum—together with equally precise coordination with the laryngeal (or supralaryngeal) sound source. In fact, articulation is so strongly linked to voluntary control that conversational laughs produced at will are more speech-like—more articulate—compared to spontaneous bursts of genuine merriment.^40^

In conclusion, our results suggest that the evolution of speech by complexification of articulation abilities has not been at the expense of our source modulation abilities. On the contrary, humans appear to have an unusually developed capacity for voluntary source modulation rivaled by only a few other animal groups, such as some marine mammals and song birds that are vocal production learners, whereas the capacity for vocal control is comparatively limited in other primates.^27^^,^^41^ How did we humans evolve these exceptional source modulation abilities? We propose two, clearly non-exclusive, scenarios: (1) Source modulation abilities could have predated speech and evolved under selection pressures to allow for faking or exaggerating affect and motivation via volitional modulation of nonverbal vocalizations, singing, and mimicry of animal vocalizations for hunting and foraging. The progressively enhanced source control required by these complex nonverbal vocal behaviors could have opened the way for optimized speech intelligibility.^27^ (2) Alternatively, selection pressure to control the laryngeal source during speech evolution may have in turn increased human volitional modulation abilities over source-related vocal parameters and led to our ability to control the production of such a wide diversity of nonverbal vocalizations. In any case, speech, singing, and nonverbal vocalizations now occupy different regions within this expanded acoustic space; these regions are presumably optimal given the functions of each type of vocal behavior. Nonverbal vocalizations existed long before speech and continue to function to communicate affective and motivational states in our species.^34^^,^^35^^,^^36^ Singing operates under loose aesthetic constraints,^42^ and at present it would be premature to speculate about the vocal features that are most desirable across all musical traditions. Finally, speech, even though it can clearly also communicate emotion and motivation, is optimized for efficient encoding of semantic information by virtue of being metabolically cheap to produce and decode, fast, and relatively robust to noise in the auditory channel.^15^ We suggest that future work should attempt to contrast this human vocal space to that of other non-human mammals in order to fully reveal continuities and discontinuities in human use of the acoustic potential offered by the mammalian vocal apparatus.

### Limitations of the study

While this appears to be the first systematic and large-scale acoustic comparison of speech with both singing and nonverbal vocalizations in human adult voices, the present study has several limitations that should be addressed in future research. Further increasing the breadth and quality of collected audio will be key to confirming and generalizing our results. For example, it will be interesting to sample both non-tonal and tonal languages more evenly (we had only five tonal languages) to compare their melodic signatures. On the other hand, we do not expect tonality to make much difference in terms of how speech compares to nonverbal vocalizations because the typical range of *f*_o_ variation in lexical tones is relatively narrow,^30^^,^^31^ and because tonal languages are not expected to include a higher proportion of nonlinear phenomena compared to non-tonal languages. More diverse and fully *a cappella* singing samples can provide more power for future analyses, calling for collection and release of extensive cross-cultural corpora.^24^ It would also be useful to extend the analysis to often non-phonated, but heavily articulated vocal behaviors such as beat-boxing, and to analyze infants’ and children’s voices, as in this study we focused exclusively on adults. Indeed, a thorough analysis of the developmental changes in verbal and nonverbal vocal behaviors will greatly contribute to the evolutionary considerations discussed above.

Intermediate forms bridging the three vocal domains, such as interjections (bridging speech/nonverbal) and vocal chants (bridging speech/singing), also deserve a more fine-grained analysis. Another potentially important distinction is between spontaneous and volitional vocal production, with the prediction that spontaneous affective bursts, even if they are partly verbalized as in expletives, may contain more nonlinear vocal phenomena and source variability than their volitional, more speech-like counterparts.^43^ Finally, it is important to note that some of the most unusual vocalizations in our collection (e.g., animal imitations or throat singing) are rare in everyday life not necessarily because of physiological constraints on vocal production, but rather because they are often socially irrelevant, considered inappropriate, and/or require extensive practice to master. The reported acoustic space could become wider if we asked participants, especially trained vocalists, to push their vocal flexibility to the extreme, for example, producing disturbing sounds outside the socially and/or aesthetically acceptable boundaries. For instance, male singers in our corpus reached at most 860 Hz (A5) in their voice fundamental frequency, but considerably higher frequencies can be produced in the whistle register. In other words, our data are based on what people typically do with their voices in non-experimental contexts, but exceptional individuals can certainly push the boundaries of the human vocal space even further.

## STAR★Methods

### Key resources table

**Table:** 

REAGENT or RESOURCE	SOURCE	IDENTIFIER
**Software and algorithms**		

R version 4.2.2	R Core Team, 2022^44^	http://www.r-project.org/
R package soundgen	Anikin^5^	https://cran.r-project.org/package=soundgen
R package brms	Bürkner^45^	https://cran.r-project.org/package=brms
R package randomForest	Liaw & Wiener^46^	https://cran.r-project.org/package=randomForest

**Deposited data**		

Original recordings, datasets, and analysis scripts		https://osf.io/a6bw5/

### Resource availability

#### Lead contact

Further information and requests should be directed to the lead contact, Andrey Anikin (andrey.anikin@lucs.lu.se).

#### Materials availability

This study did not generate new unique reagents.

### Experimental model and study participant details

#### Audio

We collected and analyzed 1745 audio recordings containing about 2 h 17 min of speech, singing, and nonverbal vocalizations (Table 1). We attempted to make the sample as representative as possible, while keeping the total amount of audio manageable for the purposes of high-quality, manually verified acoustic analysis.

Speech recordings (*n* = 616, mean duration 5.9 s, range [0.8, 33.5]) were obtained from 23 languages (Amharic, Arabic, Burmese, English, Greek, Hadza, Iban, Japanese, Javanese, Kannada, Khmer, Matlatzinca, Paunaka, Portuguese, Russian, Slovenian, Sundanese, Swahili, Swedish, Telugu, Thai, Yoruba, and Zulu) belonging to a wide range of language families (Indo-European, Sino-Tibetan, Afro-asiatic, Niger-congo, Austronesian, Dravidian, Japanonic, Arawakan, Oto-Manguean, Papuan, and Kra-Dai). Five of these languages are tonal: Burmese, Matlatzinca, Thai, Yoruba, and Zulu. The language samples were obtained from eight open-source speech databases (OpenSlr, VoxForge, VoxCeleb, ELRA, ELDP, Voices.com, AudioSet, Clarin.eu) and from the authors’ unpublished archives of speech recordings. Non-neutral speech (*n* = 200, mean duration 2.6 s, range [0.6, 32.5]) consisted of four categories: emotional speech (speech conveying a specific emotion: 66 samples^47^); oratory (speech aimed at persuasive public speaking: 13 samples from publicly available speeches delivered by internationally renowned orators such as A. Hitler, M. L. King, M. Thatcher, and M. Yousafzai); gender imitation (96 recordings of 15 female and 17 male adults in their normal voice and while sounding as masculine and feminine as possible^48^); pet-directed speech (25 samples from 13 female and 12 male adults speaking to images of dogs^49^). Pet-directed speech is similar to infant-directed speech, usually characterized by higher pitch and slower tempo than adult-directed speech.^50^

Nonverbal vocalizations (*n* = 969, mean duration 2.2 s [0.4, 26.9]) were likewise selected to have at most a few recordings per speaker. Whenever possible, we took a stratified random sample from published corpora.^51^^,^^52^^,^^53^^,^^54^^,^^55^^,^^56^ A special subcategory of nonverbal vocalizations comprised 60 animal imitations (ranging from birds and cetaceans to human babies) recorded at the IBAC conference in Brighton, 2019.

Singing samples (n = 160, mean duration 14.9 [3.9, 30.6]) were selected from a variety of musical genres (Table 1). We prioritized a cappella singing, but some recordings included relatively unobtrusive instrumental parts; the main inclusion criterion was the feasibility of manually controlled pitch tracking. Particularly unusual vocal samples, such as traditional throat singing and performances of vocal improvisors like Fatima Miranda and Demetrio Stratos,^57^ were placed in a separate subcategory of “unconventional singing”.

### Method details

#### Pitch analysis

Contours of fundamental frequency (*f*_o_) were extracted with a step of 25 ms and manually corrected using the *pitch_app* interactive environment in R package *soundgen 5*. The contours were then summarized with the *pitchDescriptives* function, including measures of average *f*_o_ (mean, median, minimum, maximum), its variability (standard deviation and range in Hz and semitones, coefficient of variability), the rate of change (average and maximum slope and absolute slope), the number of inflections per second, the proportion of voiced frames, etc. All these predictors were calculated on intonation contours after two different degrees of low-pass filtering or smoothing using Gaussian filters with a central frequency of 10 Hz and 1 Hz. The threshold of 10 Hz corresponds to the boundary between voluntary frequency modulation (e.g., a rapid vibrato) and uncontrolled vocal tremor.^7^ It preserves rapid intonation swings that are perceptually relevant, while mitigating the impact of any measurement errors that may have remained undetected even after manual correction. A cutoff of 1 Hz preserves only slower intonation patterns. In addition to low-pass filtering of frequency modulation, we applied an amplitude threshold when calculating the number of inflections: two putative inflection points had to be separated by at least 20 cents (0.2 semitones). This cutoff is still above the resolution of pitch perception (just noticeable differences in the voice-typical frequency range can be as low as 10 cents^22^), ensuring that the detected inflections are clearly audible and thus perceptually relevant, and at the same time it is high enough to guard against counting tiny “false” inflections caused by measurement error.

#### Formant analysis

For speech, we used the publicly available and extensively verified formant measurements of speech from Hillenbrand et al.,^10^ who measured the frequencies of the first four formants in 1668 recordings from 139 speakers of American English (12 vowels from each speaker). Using formant measurements from our own speech recordings produced a similar distribution in F1-F2 space, but Hillenbrand’s dataset is widely known and validated. Furthermore, the vertices of the vowel triangle in F1-F2 space are fairly universal across languages, which mostly differ in the number of vowel categories between them. Therefore, the American English vowel space is perfectly adequate as a map of human vowels in general if we are mostly interested in the area it covers, rather than in the precise vowel categories. Formant frequency measurements in Hillenbrand’s dataset were speaker-normalized by multiplying them by the ratio of apparent vocal tract length (estimated with the regression method^58^) to 17 cm. This is a way to normalize formant frequency measurements, making the resulting vowel space independent of the vocal tract length and, thus, body size of the speaker. One rater (AA) manually annotated stable vowel-like regions in nonverbal vocalizations and measured the first four formant frequencies in these regions using the *formant_app* interactive environment in *soundgen 5*. A vowel-like region was defined as a voiced part of a vocalization that had detectable and relatively stable formants and perceptually resembled a vowel produced with an open mouth and without strong nasalization. If a vocalization contained multiple stable regions corresponding to perceptually different vowels, all of them (in practice never more than three) were analyzed. It is challenging to measure formants in high-pitched vocalizations, such as screams and some laughs, because harmonics are too sparse, and *f*_o_ may lie above the first formant. Out of 969 nonverbal vocalizations, at least one vowel-like segment was analyzed in 660 vocalizations (for a total of 1126 measurements), while 309 vocalizations (32%) were omitted from formant analysis as intractable. To check the quality of this analysis, a second rater (VCP) independently measured F1-F4 in 200 randomly selected nonverbal vocalizations, in the same regions that were annotated by AA. Outliers among the vocalizations analyzed by only one rater (>3 SDs above or below the mean value for speaker-normalized F1 and/or F2) were likewise verified by both raters. Non-trivial disagreements were discussed and resolved, while for the rest of sounds the measurements by both raters were averaged. The Pearson’s correlation of the two raters’ measurements of F1 and F2 was *r* = .78 before resolving the difficult cases and *r* = .97 after resolving them.

Formant frequencies were not measured in the recordings of singing. Singing imposes constraints on articulation and may be accompanied by voluntary changes in vowel quality due to a need to match a formant with *f*_o_ or its harmonic, cluster several resonances into a so-called singer’s formant,^59^ open the mouth wide to improve voice projection,^13^ or otherwise accommodate enunciation to the requirements of the singing genre. However, such deviations from speech-typical formant configurations were not the focus of this study, and we also judged it to be technically impossible to obtain reliable formant measurements in many singing samples.

#### Modulation spectrum analysis

The modulation spectrum of a sound was calculated as the magnitude of the two-dimensional Fourier transform of its spectrogram (see Figure 3 for illustrative description). This transformation simultaneously captures the change along the time dimension (temporal modulation) and frequency dimension (frequency modulation). A separate modulation spectrum was obtained for each of 1745 recordings with a Gaussian 50 ms window and step 25 ms, averaging modulation spectra over every 10 s for recordings longer than 10 s. All modulation spectra from one category (speech, singing, or nonverbal vocalizations) were then averaged. Because the dimensionality of a modulation spectrum depends on the sound’s duration, the matrices were interpolated to the same size. This was performed automatically by the *modulationSpectrum* function in *soundgen*^5^ by placing all sounds from a particular category into a separate folder and executing the function once for each folder.

To compare speech with nonverbal vocalizations and singing, we took log-ratios of the average modulation spectra per category. The absolute values of log-ratios depend strongly on the normalization procedure, including the method of normalizing the recordings (here, to the same peak amplitude), the modulation spectrum of each recording (here, no normalization), and the aggregate modulation spectra per category (here, normalized to range from 0 to 1). Therefore, although the color coding in Figure 4 is aligned with zero in the middle of the blue-white-red gradient, there is really no natural zero, or a point at which two categories (e.g., speech and nonverbal vocalizations) have objectively the same average value in a particular pixel of the modulation spectrum matrices. Instead, the difference between spectra is best interpreted as a continuum showing the relative prevalence of particular modulation frequencies per category, and we do not find it meaningful to test the significance of pixel-by-pixel differences between categories, although this is sometimes reported (e.g.,^24^).

### Quantification and statistical analysis

We built two Bayesian mixed models to compare four pitch characteristics (Figure 1A) and the prevalence of nonlinear vocal phenomena (Figure 1C) in neutral speech, non-neutral speech, singing, and nonverbal vocalizations. Pitch descriptives were analyzed with a multivariate Gaussian model predicting each outcome as a function of speaker sex, the effect of which was allowed to vary across the main four categories as well as subcategories (e.g., musical genre or the type of nonverbal vocalization, as listed in Table 1). Fitted values were then calculated for each main category and sex and summarized as the median of posterior distribution and 95% credible interval. Nonlinear vocal phenomena were analyzed by calculating the proportion of voiced frames in each recording affected by each type of nonlinearity. These proportions were then modeled with a zero-one-inflated beta distribution^60^ as a function of category interacting with the type of nonlinear phenomenon. Two distributional parameters depended on the same predictors, namely the proportion of zero-or-one inflation probability (*zoi*) and the conditional-one probability (*coi*). Both models were fit with R package *brms*^45^ with mildly informative conservative priors.

In addition, a Random Forest classifier^61^ implemented in R package *randomForest*^46^ was used to investigate how well the main categories could be discriminated from pitch descriptives. This is a powerful method for performing classification with numerous predictors that may have nonlinear and interactive effects. The dataset was stratified by category, ensuring that underrepresented categories (e.g., singing) were adequately represented in the training sample. Out-of-bag accuracy estimates were then averaged across the outcome categories as an overall measure of classification accuracy.

## Data Availability

• All datasets for data analysis are provided as electronic supplements (https://osf.io/a6bw5/). We also included all non-copyrighted audio; the rest can be shared upon request for research purposes. • R scripts for data analysis are provided as electronic supplements (https://osf.io/a6bw5/). • Any additional information required to reanalyze the data reported in this paper is available from the lead contact upon request.
